# RGB1 Regulates Grain Development and Starch Accumulation Through Its Effect on *OsYUC11*-Mediated Auxin Biosynthesis in Rice Endosperm Cells

**DOI:** 10.3389/fpls.2021.585174

**Published:** 2021-03-31

**Authors:** Dongping Zhang, Minyan Zhang, Jiansheng Liang

**Affiliations:** ^1^Key Laboratory of Molecular Design for Plant Cell Factory of Guangdong Higher Education Institutes, Department of Biology, Southern University of Science and Technology, Shenzhen, China; ^2^Jiangsu Key Laboratory of Crop Genetics and Physiology/Co-Innovation Center for Modern Production Technology of Grain Crops, Key Laboratory of Plant Functional Genomics of the Ministry of Education, Yangzhou University, Yangzhou, China

**Keywords:** auxin, grain development, OsNF-YB1, *OsYUC11*, RGB1, rice, starch accumulation

## Abstract

RGB1, a subunit of heterotrimeric G protein, plays important roles in regulating grain size and weight of rice. However, the molecular mechanisms underlying controlling grain filling process by G protein are still largely unclear. In the present study, we show that RGB1 controls not only the grain size but also the grain filling process. Knock-down of *RGB1* significantly delayed grain development and reduced starch accumulation and grain weight, which was closely related to the delayed and the lower expression of genes encoding sucrose metabolism and starch biosynthesis related enzymes during grain filling stage. Suppression of *RGB1* expression also resulted in the lower auxin content in grains, which was correlated with the lower expression of *OsNF-YB1* and *OsYUC11* during grain filling stage. Further biochemical evidence showed that *OsYUC11* expression was under control of OsNF-YB1 by its interaction with promoter of *OsYUC11*. Taken together, we propose that RGB1 controls rice grain development and grain filling process by changing auxin homeostasis in endosperm cells. OsNF-YB1, which acts as a key downstream effector of RGB1, interacts directly with the promoter of *OsYUC11* and stimulates the *OsYUC11* expression, thereby regulating auxin biosynthesis and starch accumulation and grain size.

## Introduction

The rice grain weight is determined by both the grain sink size (numbers and size of endosperm cells) and the physiological and biochemical activities (sucrose metabolism and starch biosynthesis) of the endosperm cells for cereals (Liang et al., 2001). The rice grain is composed of the embryo and the endosperm, which is enclosed by a thin seed coat and covered by the spikelet hull (the husk), which physically restricts the size of caryopsis. The endosperm, which is the major sites storing starches and other nutritious compounds, occupies the bulk of the caryopsis. In this sense, the size and weight of the mature grain are mainly determined by the shape and size of the spikelet hull and the filling degrees of caryopsis (mainly endosperm), both of which varied greatly and controlled by different genetic and environmental factors. Rapid advances in rice functional genomics and the advent of next generation sequencing technologies have led to the recent cloning of a series of quantitative trait loci (QTLs)/genes to control grain size (length, width, thickness) and grain weight (see review by Li et al., 2018a, b; Supplementary Table 1). However, the underlying molecular mechanism regulating the grain development and grain filling processes remains elusive.

Heterotrimeric G protein (hereafter G protein)-mediated signal transduction pathway is considered as one of the most important signaling mechanisms that regulates various important physiological and molecular processes both in animals and plants (Jones and Assmann, 2004; Urano et al., 2013; Urano and Jones, 2014). In general, G protein is composed of three subunits, Gα, Gβ, and Gγ. Several researches showed that the Gβ and Gγ works as a single functional subunit (dimer) to regulate organ size (Urano et al., 2013). Rice genome encodes one Gα (RGA1), one Gβ (RGB1), and five Gγs (RGG1, RGG2, GS3, DEP1/qPE9-1, and GGC2), and evidence have shown all these G protein subunits play important roles in regulating rice grain size (Ashikari et al., 1999; Fujisawa et al., 1999; Huang et al., 2009; Zhou et al., 2009; Mao et al., 2010; Trusov et al., 2012; Liu et al., 2018; Sun et al., 2018; Zhang et al., 2019). RGA1 and two Gγ subunits, GGC2 and DEP1/qPE9-1, positively regulate rice grain size, whereas other three other Gγ subunits, RGG1, RGG2, and GS3, play opposite roles in grain size regulation (Oki et al., 2005; Huang et al., 2009; Zhou et al., 2009; Mao et al., 2010; Liu et al., 2018; Sun et al., 2018). Interestingly, both *RGB1*-knockdown lines and *RGB1*-overexpressing lines show smaller rice grain size as compared with the wildtype (Utsunomiya et al., 2011; Liu et al., 2018; Sun et al., 2018). A possible explanation is that Gγ and Gβ function as a dimer and their roles in regulating rice grain size depends on competitively coupling of Gβ with different Gγ subunits. Of course, other factors cannot be ruled out, such as MADS-domain transcription factor, BR transcription factor, BES1, etc. (Liu et al., 2018; Zhang et al., 2018).

Although G proteins play important roles in regulating grain size, the molecular mechanisms underlying controlling grain filling process by G protein are still largely unclear. In the present study, we provide both genetic and biochemical evidence to show that, RGB1 controls not only the grain size but also the grain filling process. Knock-down of *RGB1* significantly delayed grain development and reduced starch accumulation and grain weight, which was shown to be closely related to the delayed and the lower expression of genes encoding sucrose metabolism and starch biosynthesis-related enzymes during grain filling stage. We further showed that the lower auxin levels in the grains of the *RGB1*-knockdown lines are due to the lower expression of *OsYUC11* controlled by OsNF-YB1, a transcript factor. Exogenous auxin (NAA) application can partially recover the grain starch accumulation through stimulating the expression of sucrose metabolism and starch biosynthesis related genes.

## Materials and Methods

### Generations of Knock-Down Mutants Using RNA Interfering Technology

To generate RGB1 RNAi transgenic lines, a gene fragment of RGB1 was amplified from Nipponbare cDNA. A hairpin structure with two inverted repeat fragments was subsequently constructed and transferred into the plant binary vector pTCK303, expressed under the control of the maize ubiquitin promoter (Wang et al., 2004). The construct was transformed into the *Agrobacterium* strain EHA105 and used for plant transformation as described previously (Chen et al., 2014). The DNA fragments embracing the targets of the plant transformants were amplified for sequencing.

### Plant Growth Conditions and Treatments

The experiment was carried out at the farm of Yangzhou University (32°30′N, 119°25′E) during the rice (*Oryza sativa*) growing season, and in Hainan in winter seasons. At the heading stage, 200 uniformly growing and headed panicles (one to two panicles per plant) were chosen, and spikelets on the selected panicles with the same flowering date were labeled for each cultivar/line. The flowering date and position of each spikelet on the labeled panicles were recorded. Approximately 30 labeled panicles were sampled each time from flowering to maturity. Half of the sampled grains were frozen in liquid nitrogen for at least 2 min and then stored at −80°C for further analyses. The other half of the grains were dried at 80°C for approximately 72 h to a constant weight and used for starch analyses.

For exogenous NAA application, rice plants growing in the plastic pots (three hills per pot) under open field conditions were used. Each pot (0.6 m in height, 0.5, and 0.3 m in diameter at the top and bottom, respectively), was filled with sandy loam soil that contained the same nutrient contents as the field soil. The sowing date and cultivation were the same as those for the field experiment. After flowering, 10 μM NAA was sprayed at a rate of 5 ml per pot on the top of the plants (spikes) every 5 days until 30 DAF. The solutions contained ethanol and Tween-20 at final concentrations of 0.1% (v/v) and 0.01 (v/v), respectively. Control plants were sprayed with the same volume of deionized water containing the same concentrations of ethanol and Tween-20.

### Gene Expression Analysis

Total RNA was extracted from the grains or young panicle using the RNAprep Pure Plant Kit (Tiangen). The HiScript II Q Select RT SuperMix (Vazyme) was used for cDNA synthesis. The transcript levels of each gene were measured by qRT-PCR using a 7500 Real-Time PCR System (ABI) with PowerUp^TM^ SYBR^®^ Green Master Mix (Thermo Fisher Scientific). Gene expression was quantified during the logarithmic phase using the expression of the housekeeping gene Ubq (LOC_Os03g13170) as an internal control. Three biological replicates were performed for each experiment.

### Hormone Quantification

The IAA level was determined by Zoonbio Biotechnology Co., Ltd. Samples (0.5 g) were ground in a pre-cooled mortar that contained 5 ml of extraction buffer composed of isopropanol/hydrochloric acid. The extract was shaken at 4°C for 30 min. Then, 10 ml of dichloromethane was added, and the sample was shaken at 4°C for 30 min and centrifuged at 13,000 rpm for 5 min at the same temperature. The organic phase was dried under N_2_ gas, and the pellet was dissolved in 150 μl of methanol (0.1% methane acid) and filtered with a 0.22 μm filter membrane. The purified product was then subjected to high-performance liquid chromatography-tandem mass spectrometry (HPLC-MS/MS) analysis. HPLC analysis was performed using a ZORBAX SB-C18 (Agilent Technologies) column (2.1 mm × 150 mm; 3.5 mm). The mobile phase A solvents consisted of methanol/0.1% methanoic acid, and the mobile phase B solvents consisted of ultrapure water/0.1% methanoic acid. The injection volume was 2 μl. MS conditions were as follows: the spray voltage was 4,500 V; the pressures of the air curtain, nebulizer, and aux gas were 15, 65, and 70 psi, respectively; and the atomizing temperature was 400°C.

### Protein Blot Analysis

Rice grains (from 5 to 25 DAF) were homogenized in TEDM buffer (20 mM Tris/HCl, pH 7.5, 1 mM DTT, 5 mM EDTA, and 10 mM MgCl_2_) containing a complete protease inhibitor cocktail (Roche). The homogenate was centrifuged at 6,000 g for 10 min at 4°C to remove cellular debris, and the supernatant was clarified by centrifugation at 12,000 g for 10 min at 4°C. The soluble proteins were separated by SurePAGE (4–12%, Genscript) and blotted onto a PVDF membrane (Millipore). Anti-HSP82 antibodies were used as a loading control (Beijing Protein Innovation).

### Transcriptome Sequencing

For transcriptome sequencing, total RNA was extracted from the grains at 5 and 10 DAF, using the RNAprep Pure Plant kit (Tiangen). RNA integrity and quantity were determined with an Agilent 2100 Bioanalyzer according to the manufacturer’s protocol. Enrichment of mRNA from total RNA, complementary DNA synthesis, and construction of the library were performed at Vazyme Biotech. The total libraries were sequenced using an Illumina HiSeq X Ten platform. The raw reads were filtered by removing reads with adapters, reads in which unknown bases were >5%, and low-quality reads. The FASTQC program was used to assess the quality of the clean reads, which were then aligned to the rice reference genome Release 7 of the MSU Rice Genome Annotation Project using TOPHAT v.2.1.0 (Trapnell et al., 2012). CUFFLINKS v.2.1.1 was then used to normalize and estimate the gene expression level according to fragments per kilobase of transcript per million reads (FPKM, Mortazavi et al., 2008). The differentially expressed genes (DEGs) were also calculated using CUFFLINKS v.2.1.1 at a significance level of *q* < 0.05 and the absolute value of log2 ratio ≥1.

### Electrophoresis Mobility Shift Assay (EMSA)

The flag-OsNF-YB1 protein was expressed using Gzl Custom Cell Free Protein Expression Kit (GZL bioscience). DNA probes in a length of 59 nt were commercially synthesized by GENEWIZ Biological Technology and labeled with an EMSA Probe Biotin Labeling Kit (Beyotime, Cat No. GS008). DNA binding was performed in a 10-L reaction volume containing EMSA/Gel-shift binding buffer (Beyotime), 2 nmol biotin-labeled probe, and 5 nmol purified recombinant protein. Non-labeled DNA oligos were used as competitor. Recombinant protein was pre-incubated with the EMSA/Gel-shift binding buffer for 20 min at 25°C prior to the addition of the biotin-labeled probe and further incubated at 25°C for 20 min. A 6% (W/V) polyacrylamide gel was pre-run for 30 min, and then the binding reaction is subjected to gel electrophoresis. The DNA probes were then transferred to a charged nylon membrane (Beyotime), detected by streptovidin-HRP (Beyotime), and finally visualized using enhanced chemiluminescence (Pierce).

### Statistical Analysis

Data are presented as mean ± *SD*. The SPSS16.0 software was used for all statistical analysis. Statistical significance was determined by independent biological samples student’s *t*-test for comparison of two groups and one way ANOVA for comparison of three or more groups. Differences were considered statistically significant when *P* < 0.05. *P-*values are indicated by “*” when *P* < 0.05 or “**” when *P* < 0.01.

## Results

### *RGB1* Positively Regulates Both Grain Size and Grain Filling

**FIGURE 1:**
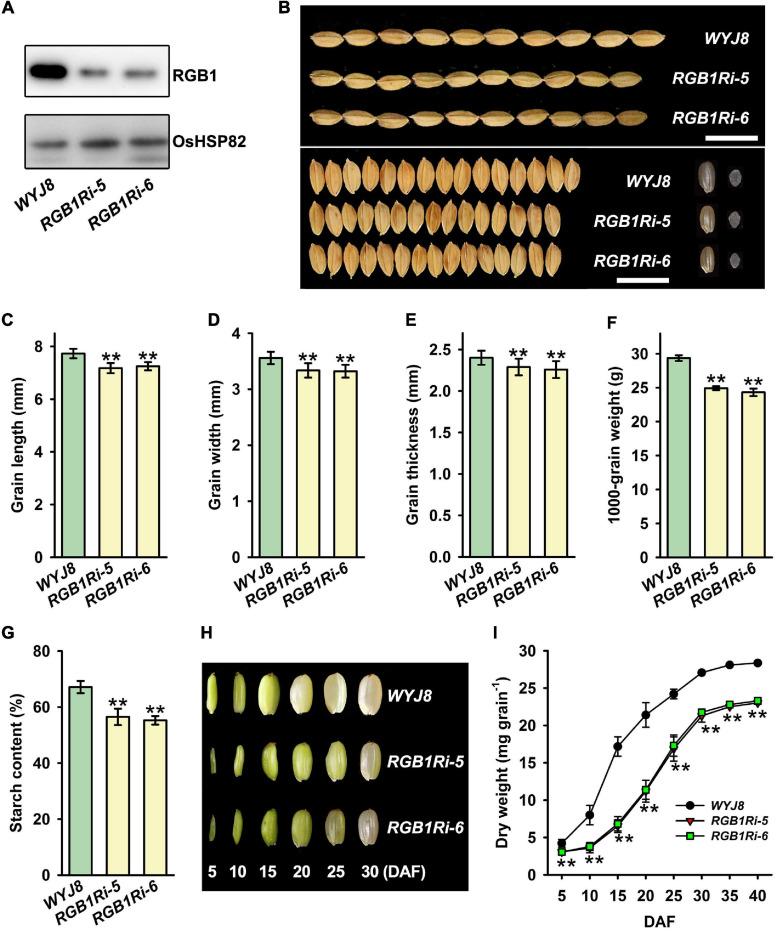
Comparison of grain size and dry weight accumulation between wildtype (*WYJ8*) plant and RGB1 RNA-interfered transgenic lines. **(A)** Immunoblot analysis of the transgenic plants. Total proteins are extracted from the panicles, and 10 μg protein was used for western blotting. **(B)** Grain morphology (size and shape). Scale bar: 15 mm. **(C–F)** Comparison of grain size and weight between wild type (*WYJ8*) and RGB1 RNA-interfered transgenic lines: **(C)** Grain length; **(D)** Grain width; **(E)** Grain thickness; **(F)** 1,000-grain weight. **(G)** The grain starch content. **(H)** The changes of caryopsis morphology at different days after flowering. **(I)** Changes in grain weight during grain-filling. Data are shown as means ± *SD*s (*n* = 15). **Means significant differences when *P* < 0.01.

Previous studies have showed that *RGB1* play important roles in regulating growth and development of rice plants (Utsunomiya et al., 2011; Sun et al., 2018). However, it remains largely unknown how the grain development and grain filling is regulated by *RGB1*. To investigate the molecular mechanisms of RGB1 regulation on grain development and grain filling, we used Wuyujing 8 (hereafter *WYJ8*) as background, to generate two *RGB1* RNA-interfered transgenic lines (hereafter *RGB1Ri-5* and *RGB1Ri-6*). Compared with *WYJ8*, the grain RGB1 protein levels were significantly lower in the *RGB1Ri-5* and *RGB1Ri-6* lines, measured by Western blot assay using an RGB1-specific antibody (Figure 1A). The final grain size, including the length, width, and thickness of grains, of two transgenic rice lines with suppressed expression of *RGB1* were markedly reduced, and consequently, the final grain weight reduced significantly, compared with *WYJ8* (Figures 1B–F), which was very similar to the phenotype observed in several other reports (Utsunomiya et al., 2011; Sun et al., 2018). Furthermore, the grain starch content of *RGB1Ri* lines was also lower than that of *WYJ8* (Figure 1G). We further tracked the caryopsis development and dry matter accumulation after flowering, and the caryopsis size and grain weight of *WYJ8* increased rapidly and maximal values were reached at 25 DAF. However, the increase of caryopsis size was obviously delayed and the dry matter accumulation of grains much slowly during the early stage of grain filling of *RGB1Ri* lines (Figures 1H,I). These results implied that *RGB1* might also be involved in regulating the grain filling processes as well as the grain size.

### *RGB1* Enhances the Expression of Genes Associated With Sucrose Metabolism and Starch Biosynthesis

To investigate how *RGB1* regulates the grain development and starch biosynthesis, the expression profiles of genes in caryopsis of *WYJ8* and *RGB1Ri-5* line at 5 and 10 DAF were analyzed, using the RNA-seq assay. Approximately 3,216 and 3,363 genes that were up- or downregulated, respectively, in *RGB1Ri-5* compared with *WYJ8*, were identified at 5 DAF. The numbers of upregulated genes at 10 DAF decreased to 2,832 and those of downregulated genes increased to 3,398, respectively (Supplementary Figure 1A). The Kyoto Encyclopedia of Genes and Genomes (KEGG) pathway enrichment analysis showed that these differently expressed genes (DEGs) were significantly enriched for “sucrose metabolism and starch biosynthesis” and “plant hormone transduction” (Supplementary Figure 1B), suggesting that *RGB1* may regulate sucrose metabolism and grain starch synthesis through manipulating hormone homeostasis and/or hormone-mediated signaling.

**FIGURE 2:**
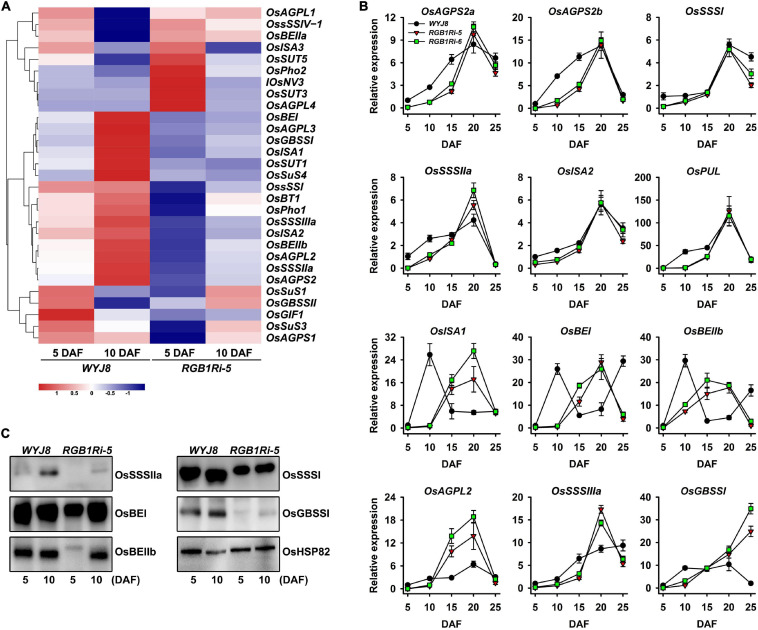
Expressional profiles of sucrose metabolism and starch synthesis related genes during grain filling stage of *WYJ8* plant and *RGB1Ri lines*. **(A)** Heat maps of differentially expressed genes (DEGs) involved in the starch biosynthesis. The maps were plotted using the value of RPKM for each gene in the different samples: blue indicates low values; red indicates high values. The indicated scale is the log2 value of the normalized level of gene expression. **(B)** qRT-PCR analysis showing the changes of genes encoding sucrose metabolism and starch biosynthesis-related enzymes during grain filling (from 5 to 25 DAF). **(C)** Western blotting assay showing the changes of the levels of several enzymes/proteins related to starch biosynthesis at the early stage of grain filling (5 and 10 DAF).

To validate the above hypothesis, we analyzed the expression patterns of DEGs for sucrose metabolism and starch synthesis (29 genes) and grain development regulation (17 genes). The results indicated that the expression of about 70% of the sucrose metabolism and starch biosynthesis-related genes and all the grain development-related genes were upregulated in *WYJ8*, compared with that in *RGB1Ri-5* line (Figure 2A). These results were consistent with our observation that *RGB1* positively regulate grain filling and starch accumulation. We further analyzed the dynamic expression patterns of these genes in grains of *WYJ8* and *RGB1Ri* lines during grain filling period using both real-time quantitative PCR and Western blotting assay. As shown in Figure 2B, the dynamic expression patterns of all 12 sucrose metabolism and starch biosynthesis-related genes could be roughly divided into three groups. *OsAGPS2a*, *OsAGPS2b*, *OsISA2*, *OsSSSI*, *OsSSSIIa*, and *OsPUL* belonged to the first group, of which their transcript levels rapidly increased after flowering, and reached their maximal levels at 20 DAF, and then decreased both in *WYJ8* and in *RGB1Ri* lines. However, the expression levels of these six genes in grains of *WYJ8* were higher than in the *RGB1Ri* lines, especially during the early stage of grain filling. The second group included *OsISA1*, *OsBE1*, and *OsBEIIb*, and the transcript levels of these three genes increased in grains of all genotypes, but the time reached maximal levels, which was different between genotypes. The maximal transcript levels of these genes in *WYJ8* grains were detected at 10 DAF, which was much earlier than those of the *RGB1Ri* lines. *OsAGPL2*, *OsSSSIIIa*, and *OsGBSSI* genes belonged to the third group, of which the transcript level in the grains of the *WYJ8* line was higher at the early stage of grain filling, whereas the transcript levels in the grains of the *RGB1Ri* lines increased rapidly after 10 DAF and kept much higher levels thereafter. The Western blotting results were well consistent with the RT-PCR results, of which the protein level of OsSSSIIa, OsBEI, OsBEIIb, OsSSSI, and OsGBSSI were much lower in grains of *RGB1Ri-5* line at the early stage of filling (Figure 2C). Taken together, these results indicate that *RGB1* play key roles in controlling grain filling through its effects on the expression of genes encoding sucrose metabolism and starch biosynthesis-related enzymes, especially at the early stage of grain filling.

### *RGB1* Stimulates Accumulation of IAA by Regulating the Expression of Auxin Biosynthesis-Related Genes

**FIGURE 3:**
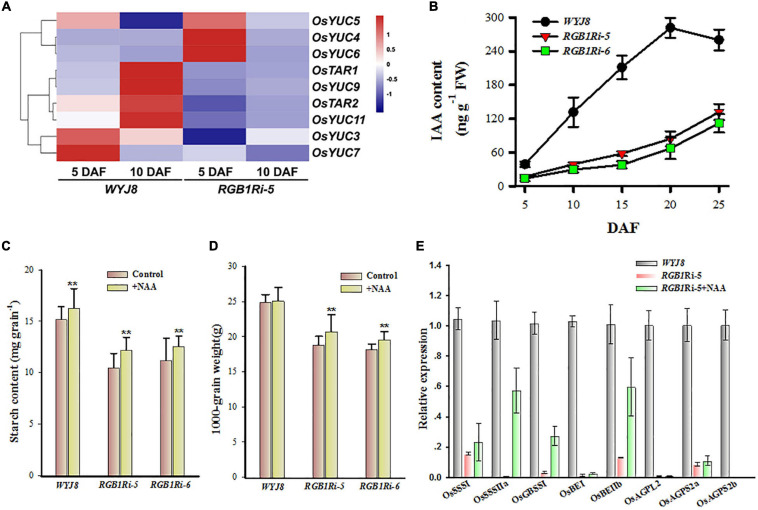
Expressional profiles of auxin biosynthesis-related genes and changes in IAA in grains of *WYJ8* plant and *RGB1Ri* line and effects of application of NAA on starch biosynthesis. **(A)** Heat maps of differentially expressed genes (DEGs) involved in auxin biosynthesis. The maps were plotted using the value of RPKM for each gene in the different samples: blue indicates low values; red indicates high values. The indicated scale is the log2 value of the normalized level of gene expression. **(B)** Changes in IAA level in rice endosperm cells of different genotypes during grain filling stage. **(C)** Effects of exogenous auxin (NAA) application on starch accumulation of grains of different genotypes. **(D)** Effects of exogenous auxin (NAA) application on grain weight of different genotypes. **(E)** Effects of exogenous auxin (NAA) application on the expression of genes related to sucrose metabolism and starch biosynthesis at 10 DAF. Data are shown as means ± *SD*s (*n* = 5). **Means significant differences when *P* < 0.01.

It is well known that grain development and sucrose metabolism and starch biosynthesis are highly regulated by both the genetic and the environmental cues. Plant hormones are involved in the regulation of many aspects of grain development, and sucrose metabolism and starch biosynthesis (Tang et al., 2009; Yang and Zhang, 2010; Hu et al., 2013; Zhang et al., 2014; Zuo and Li, 2014). Our previous study and other research groups’ results clearly showed that auxin plays important roles in these processes (Zhao, 2008; Ghorbani et al., 2011; Abu-Zaitoon et al., 2012; Zhao et al., 2013; Tamaki et al., 2015; McAdam et al., 2017). However, the molecular basis of auxin accumulation in rice grains is largely unclear. Based on the results of RNA-seq assay and the KEGG pathway enrichment analysis, we found that there were 77 DEGs involved in auxin biosynthesis and metabolism, transport, and signaling during the grain filling period (Supplementary Figure 2). Among them, six auxin biosynthesis genes were upregulated in the *WYJ8* grains at 10 DAF, compared with those in the *RGB1Ri-5* line (Figure 3A), implying that *RGB1* positively regulates sucrose metabolism and starch biosynthesis probably through controlling IAA biosynthesis in grains.

To validate this hypothesis, we measured the endogenous IAA content of grains during the grain filling period. The results showed that the *WYJ8* grain IAA content increased almost linearly after flowering and reached its maximal value at 20 DAF, whereas those of the *RGB1Ri* lines increased much slowly, especially at the early stage of grain development, and kept much lower levels throughout all experimental period (Figure 3B). Application of NAA after flowering accelerated starch accumulation and enhanced the final grain weight significantly of *RGB1Ri-5/6* lines (Figures 3C,D). NAA application also stimulated the expression of *OsSSSI*, *OsSSSIIa*, *OsGBSSI*, *OsBEIIb*, and *OsAGPS2a*, but had no effects on the expression of the other seven genes in Figure 2, compared with the no-NAA-treated *RGB1Ri-5* line (Figure 3D). All these indicate positive correlations between IAA and sucrose metabolism and starch biosynthesis and between *RGB1* expression and grain IAA level. So, it is reasonable to assume that *RGB1* stimulates grain filling, and sucrose metabolism and starch biosynthesis largely through influencing auxin biosynthesis.

**FIGURE 4:**
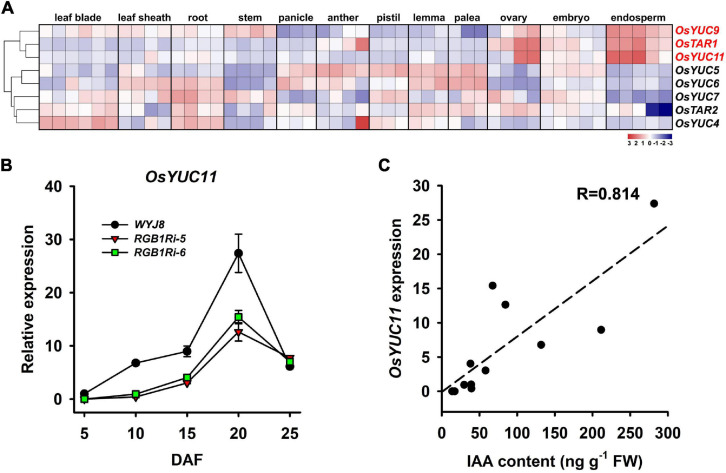
Tissue-specific expressions of *OsYUC11* and its relation to auxin content of grains**. (A)** Expression analysis of *OsYUC11* in public microarray data. The maps were plotted using the value of normalized signal intensity for each gene in the different samples: blue indicates low values; red indicates high values. The indicated scale is the log2 value of the normalized level of gene expression. **(B)** qRT-PCR analysis showing the expression of *OsYUC11* in the grains of *WYJ8* and *RGB1Ri* lines during filling stage. **(C)** The relationship between the expressional levels of *OsYUC11* and IAA content in grains.

In higher plants, the *de novo* IAA biosynthesis is *via* TAR/YUCCA pathway. Our RNA-seq analysis results, revealed that several auxin biosynthesis genes, including *YUCs* and *TARs*, were downregulated in grains of *RGB1Ri* lines either at 5 DAF or 10 DAF (Figure 3A). There is evidence that IAA plays several distinct roles at different stages of endosperm development (see review by Figueiredo and Köhler, 2018; Basunia and Nonhebel, 2019). These differing roles require strong localized control of IAA biosynthesis by specific enzymes. According to the public available expression data, three genes, *OsYUC9*, *OsTAR1*, and *OsYUC11*, are specifically and highly expressed in rice endosperm cells (Figure 4A). Our qRT-PCR results showed that the transcript levels of all three genes increased at the early filling stage of wildtype grains, whereas those in *RGB1Ri* lines kept lower at the same stage of grain filling and increased until 15 DAF (Figure 4B and Supplementary Figure 3A), suggesting their involvement in IAA biosynthesis in grains. However, among three genes, only the expression of *OsYUC11* was significantly correlated to the grain IAA levels (Figure 4C and Supplementary Figure 3B). If we considered the temporal expression patterns of *OsYUC11* combined with the results of the initiation of rapid accumulation of IAA and starch in grains of both wildtype and *RGB1Ri* lines (Figures 1I, 3B), it is reasonable to assume that various auxin biosynthesizing genes may control auxin production in different organs/tissues and the endosperm-specific *OsYUC11* gene is the most important one that is responsible for auxin biosynthesis of grains during filling stage, and that *RGB1* stimulates auxin accumulation through enhancing the expression of *OsYUC11* gene in grains.

### RGB1 Regulates Expression of *OsYUC11* Probably Through *OsNF-YB1*

**FIGURE 5:**
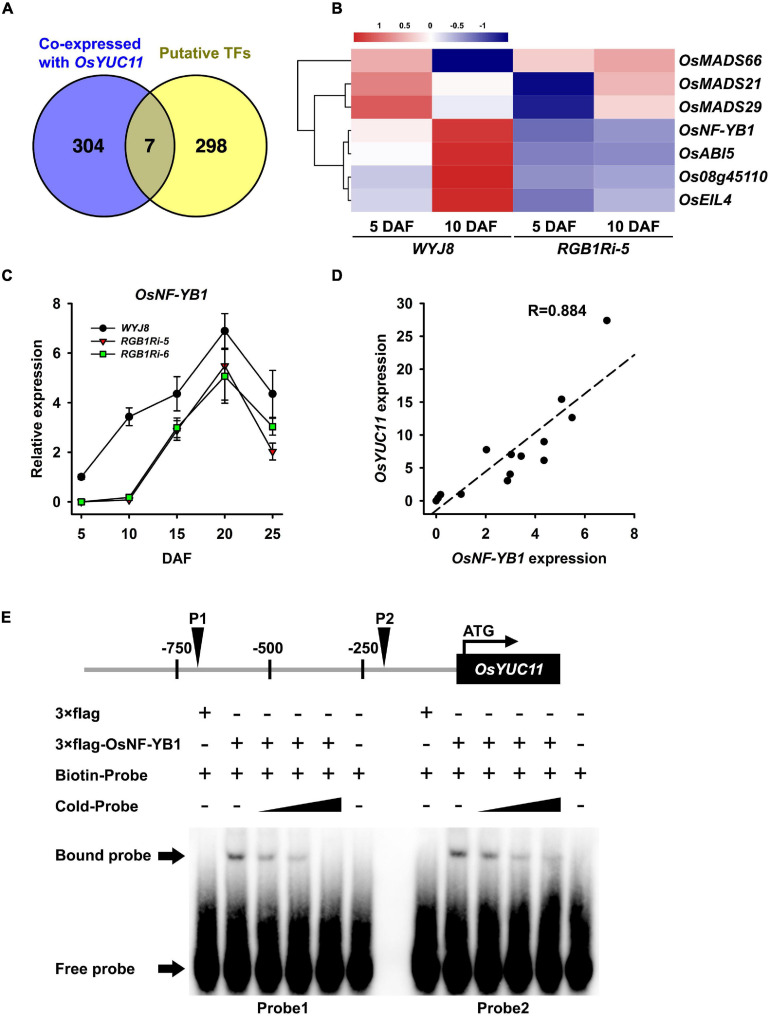
The transcript factor OsNF-YB1 directly interacts with promoter of *OsYUC11*. **(A)** Venn diagram showing number of genes included in co-expressed with *OsYUC11* and putative transcript factors which bind to the promoter of *OsYUC11*. Venn diagram has been generated using Venn (http://bioinfogp.cnb.csic.es/tools/venny/index.html). **(B)** Heat map of the expression of candidate transcript factors in *WYJ8* and *RGB1Ri* line during filling stage. **(C)** Expression of *OsNF-YB1* in *WYJ8* and *RGB1Ri* lines. **(D)** Correlation between the expression of *OsYUC11* and *OsNF-YB1*. **(E)** EMSA assay showing OsNF-YB1 binding to the *OsYUC11* promoter *in vitro*.

The results above showed that RGB1 regulates grain development and sucrose metabolism and starch accumulation probably through stimulating the expression of endosperm-specific *OsYUC11*, which enhances auxin biosynthesis in endosperm cells. However, how RGB1 regulates the expression of *OsYUC11* is still elusive. In the present experiment, we first analyzed the promoter of *OsYUC11* that might bind and regulate *OsYUC11* expression using PlantPAN 2.0 database. The results showed that there were 305 putative transcript factors, which might bind to the promoter of *OsYUC11* (Figure 5A and Supplementary Table 2). Among them, four transcript factors, *Os08g45110*, *OsNF-YB1*, *OsABI5*, and *OsEIL4*, were both co-expressed with *OsYUC11* and downregulated in *RGB1Ri* line on 5 and 10 DAF (Figures 5A,B), implying that the expression of *OsYUC11* might be regulated by these four transcript factors. We further compared the temporal expression patterns of these four transcript factors in grains of wildtype and *RGB1Ri* lines and the results showed that only the transcript levels of *OsNF-YB1* increased rapidly and were higher in the wildtype grains than those in *RGB1Ri* lines, especially at the rapid stage of grain filling (Figure 5C), which is well consistent with those of starch accumulation and grain IAA content (Figures 1I, 3B,C). Furthermore, the transcript levels of *OsNF-YB1* were positively related to those of *OsYUC11* (Figure 5D). It is reasonable to assume that *OsNF-YB1* play a key role in controlling *OsYUC11* expression, and finally the auxin content in grains. To verify this assumption, we also conducted an EMSA assay, and the results clearly showed that OsNF-YB1 was able to bind the promoter of *OsYUC11* (Figure 5E). Based on the above results, we proposed that the expression of *OsYUC11* in rice grains was under the control of *OsNF-YB1*.

## Discussion

The grain size and weight of rice are complex traits, which involves in grain development and filling process (mainly the sucrose metabolism and starch biosynthesis), and is controlled by both genetic and environmental factors. In the last decades, several research groups have provided genetic and molecular evidence that grain size and filling processes are controlled by many genes (see review by Zuo and Li, 2014; Sun et al., 2018). All these researches provide rice breeders opportunities to improve rice grain yield by manipulating these genes. However, it still has a long way to go before understanding the complex regulatory networks involving grain development and filling processes. The heterotrimeic G protein (hereafter G protein) is well known to play important roles in plant growth and development (Botella, 2012; Sun et al., 2018). Recently, several rice genes/*QTLs* encoding G protein subunits have been shown to control grain size and shape of rice, including *qPE9-1/DEP1*, *GS3*, *RGB1*, and *RGA1* (Oki et al., 2005; Huang et al., 2009; Zhou et al., 2009; Mao et al., 2010; Liu et al., 2018; Miao et al., 2018; Sun et al., 2018). *qPE9-1/DEP1* encoding a Gγ subunit, positively regulates grain size (Huang et al., 2009; Zhou et al., 2009); GS3, another Gγ subunit, negatively regulate grain size and shape (Mao et al., 2010; Sun et al., 2018); *RGB1* and *RGA1*, encoding Gβ and Gα subunit, respectively, positively regulates grain size and shape (Fujisawa et al., 1999; Utsunomiya et al., 2011; Sun et al., 2018). However, the functions and molecular mechanisms of G protein to regulate grain development and starch biosynthesis are largely unknown.

Rice grain filling is a very complicated process that involves photoassimilate translocation from photosynthetic sources (i.e., leaves and stem-sheaths), sucrose degradation, transmembrane transport, and starch synthesis in the grains (Liang et al., 2001; Lü et al., 2008; Tang et al., 2009). Approximately 20 enzymes/proteins have been reported to be involved in these biochemical processes (Zhu et al., 2003; Tetlow et al., 2004; Ohdan et al., 2005). The results of our lab and several other groups showed that sucrose synthase (SUS), invertase (INV), ADP-glucose pyrophosphorylase (AGP), soluble starch synthase (SSS) and grannuel-bound starch synthase (GBSS), branching enzyme (BE), and debranching enzyme (DBE) play key roles in the regulation of sucrose metabolism and starch biosynthesis (Liang et al., 2001; Lü et al., 2008; Tang et al., 2009; also see reviewed by Tetlow et al., 2004; Keeling and Myers, 2010; Zeeman et al., 2010). The grain-filling process is also a highly regulated process in which both genetic and environmental factors are involved. It is well known that plant hormones play important roles in grain development and filling process (Liang et al., 2001; Yang et al., 2006; Tang et al., 2009; see also review by Basunia and Nonhebel, 2019). The endosperm cell proliferation and elongation as well as the accumulation of starch and other storage compounds after flowering are of crucial importance for final grain yield and quality. Our results showed that grain size, including the length, width, and thickness of grains reduced, and as a result, the final grain weight and starch content significantly reduced in *RGB1* knock-down lines (Figure 1). This reduction was mainly due to the delay of caryopsis development and the lower starch accumulation at the early stage of grain filling (Figures 1H,I). Analyses on the expression levels of genes encoding enzymes catalyzing sucrose metabolism and starch biosynthesis showed that, in *RGB1* knock-down lines, the expression of genes encoding sucrose metabolism and starch biosynthesis was either initiated later or much lower at the early filling stage, which can well explain the results observed (Figures 1, 2) and imply that suppression of *RGB1* expression could down-regulate the expression of genes encoding enzymes catalyzing sucrose metabolism and starch biosynthesis. However, the molecular mechanisms of *RGB1* regulation on the expression of these genes are still unknown. Overexpression of *RGB1* had no effects on grain development and grain filling processes (data not shown).

Recently, several research groups have reported an accumulation of auxin immediately before the starch biosynthesis in rice grains (Abu-Zaitoon et al., 2012). Furthermore, application of exogenous auxin also had a positive effect on starch accumulation. These results suggested that auxin might involve in the regulation of starch biosynthesis in rice grains. RNA-seq assay and auxin quantitation also showed a great difference in the expression of auxin biosynthesis related genes and IAA contents in grains between *RGB1* knocking down lines and wildtype during filling stage. The expression of endosperm-specific genes for auxin biosynthesis was downregulated and endogenous IAA content significantly reduced in the grains of *RGB1* knocking down lines (Figure 3). The assumption that RGB1 involvement in the regulation of starch biosynthesis is through changing auxin homeostasis of grains was further validated according to the results of exogenous application of IAA on starch accumulation and the expression of sucrose metabolism and starch biosynthesis related genes during grain filling stage (Figure 3). Of course, the contribution of auxin transport from other parts to the auxin homeostasis in the endosperm cells could not be ruled out. In fact, there were some reports that auxin transport was also involved in the regulation of grain size (Liu et al., 2015).

Auxin biosynthesis in higher plants is catalyzed by a large number of *TARs* (encoding tryptophan aminotransferase) and *YUCs* (encoding indole-3-pyruvate mono-oxygenases) with differing patterns of spatiotemporal expression, which allows for multiple roles. Different genes may be responsible for the auxin production in different times and/or in different tissues, and therefore, play various roles in regulating plant growth and development. In rice, tissue-specific expression of these genes showed that *OsTAR1*, *OsYUC9*, and *OsYUC11* genes were expressed highly in the endosperm cells (Figure 4A), suggesting these three genes might play important roles in controlling auxin biosynthesis in grains. However, only the expression of *OsYUC11* was well correlated with the grain IAA content (Figure 4C). Based on these results, we hypothesize that *OsYUC11* were mainly and especially responsible for the auxin biosynthesis in endosperm cells of rice and other auxin biosynthesis related genes may participate in separate signaling processes.

It is clear that the delay of caryopsis development and the lower starch accumulation and grain weight in *RGB1* knocking down lines are due to the lower auxin content in grains caused by the lower expression of *OsYUC11* during grain filling stage. Furthermore, *OsYUC11* also plays a positive regulatory role in the starch biosynthesis pathway by upregulating the expression of several sucrose metabolism and starch biosynthesis related genes. Collectively, our study suggests that *OsYUC11* is of crucial importance in regulating grain development and starch biosynthesis by controlling auxin biosynthesis during grain filling stage. In eukaryotes, transcription of genes is regulated by various transcription factors. Our results showed that *OsYUC11* promoter may interact with several families of transcript factors, including MADS, MYB, CCAAT, etc. (Supplementary Table 1), suggesting that the regulation of *OsYUC11* expression is very complicated and involves signaling networks. However, if we considered the results of RNA-seq analysis that showed the differences of the expression of various transcription factors between *RGB1Ri* line and *WYJ8* plant during grain filling stage, and the results of tissue-specific analysis of gene expression of these transcript factors, it is reasonable to assume that *OsNF-YB1* might involve in the regulation of the expression of *OsYUC11*. Our present results provide biochemical evidence to support the conclusion that *OsNF-YB1* was crucially important in regulating *OsYUC11* expression and, finally, the auxin content in grains. *OsNF-YB1* has been reported to activate the expression of sucrose transporters and *waxy* gene and, finally, regulates the endosperm development. Knockout of Os*NF-YB1* led to defective grains with chalky endosperms and significantly decreased grain weight (Bai et al., 2016; Bello et al., 2018). Our results provide a new insight that Os*NF-YB1* regulates grain development not only through directly activating the sucrose metabolism and starch biosynthesis genes but also through controlling the auxin accumulation.

**FIGURE 6:**
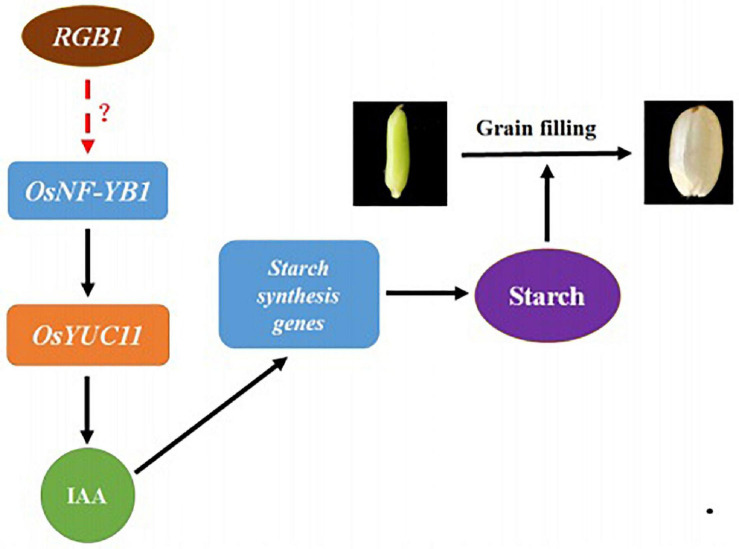
A working model on RGB1 regulation on grain filling. RGB1 may positively regulate expression of transcription factor *OsNF-YB1* through unknown mechanism(s), which activates the *OsYUC11* transcription by interacting with its promoter, and leads to an increase in auxin level. The increased auxin then stimulates the expression of sucrose metabolism and starch biosynthesis-related genes in endosperm cells, as a consequence, the biosynthesis of starch and grain filling.

In summary, according to our present results, a working model is proposed to illustrate the roles of RGB1 in regulating grain development and grain filling process. RGB1 may positively regulate expression of transcription factor *OsNF-YB1* through unknown mechanism(s), which activates the *OsYUC11* transcription by interacting with its promoter, and leads to an increase in auxin level. The increased auxin then stimulates the expression of sucrose metabolism and starch biosynthesis-related genes in endosperm cells and, as a consequence, the biosynthesis of starch and grain filling. However, the mechanism underlying how RGB1 regulates the expression of *OsNF-YB1* remains to be further studied (Figure 6).

## Data Availability Statement

The original contributions presented in the study are publicly available. This data can be found here: https://www.ncbi.nlm.nih.gov/geo/, GSE169377.

## Author Contributions

All authors listed have made a substantial, direct and intellectual contribution to the work, and approved it for publication.

## Conflict of Interest

The authors declare that the research was conducted in the absence of any commercial or financial relationships that could be construed as a potential conflict of interest.
