# Supplementary material for: RGB1 Regulates Grain Development and Starch Accumulation Through Its Effect on OsYUC11-Mediated Auxin Biosynthesis in Rice Endosperm Cells
Source: Front Plant Sci. 2021 Mar 31;12:585174. doi: 10.3389/fpls.2021.585174 (PMC8045708; doi:10.3389/fpls.2021.585174)
Supplement: Supplementary file 1 [file Table_1.DOCX]

Table S1 List of genes/QTLs involved in regulating rice grain size and grain filling

| **Gene/QTL name** | **Protein category encoded** | **Major functions** | **References** |
| --- | --- | --- | --- |
| **Grain size/weight** |  |  |  |
| *GS3* | Homolog of *Arabidopsis* G protein γ subunitn | Cell proliferation | Fan, et al. (2006) |
| *GL3.1* | Protein phosphatase kelch family-Ser/threonine protein phosphatase | Cell proliferation | Qi, et al. (2012) |
| *An-1* | A basic helix-loop-helix protein | Cell proliferation | Luo ,et al. 2013 |
| *GS2* | Rice growth-Regulating Factor 4 (OsGRF4) | Cell expansion | Hu, et al. (2015) |
| *GL2* | Unknown protein | Cell expansion | Che, et al. 2015 |
| *GW2* | A RING-type protein with E3 ubiquitin  ligase activity | Cell proliferation | Song, et al. 2007 |
| GW5 | A plasma membrane-associated protein  with IQ calmodulin-binding motifs | Cell proliferation | Liu, et al. (2017) |
| *GS5* | Putative serine carboxypeptidase | Grain width and grain filling | Li, et al. (2011) |
| *RGB1* | Rice G protein β subunit | Cell proliferation | Utsunomiya, et al. (2011) |
| *GL7/GW7* | A protein homologous to *Arabidopsis thaliana* LONGIFOLIA protein | Cell elongation | Wang, et al. (2015) |
| *DEP1/qPE9-1* | Homolog of Arabidopsis G protein γ subunit | Cell proliferation | Huang, et al. 2009; Zhou et al. 2009 |
| *PGL1* | bHLH protein | Cell expansion | Heang and Sassa, 2012 |
| *SG1* | An unknown protein | Cell expansion | Nakagawa, et al. 2012 |
| *MIS2* | Encodes a Receptor-like KinaseCRINKLY4 (CR4) | Cell proliferation and expansion | Chun, et al. 2020 |
| *RBG1* | Unknown protein | Cell division | Lo, et al. 2020 |
| *TGW6* | IAA-glucose hydrolase | IAA biosynthesis | Ishimaru, et al. 2013 |
| *BG1* | Membrane-localized protein | Cell proliferation and expansion | Liu, et al. 2015 |
| *XIAO* | A putative LRR receptor-like kinase | Cell proliferation | Jiang, et al. 2012 |
| *GSN1* | A mitogen-activated protein  kinase phosphatase (OsMKP1) | Cell proliferation | Guo, et al. 2018 |
| *OsAGO17* | A putative AGO protein | Cell elongation | Zhong, et al. 2020 |
| *GLW7* | A plant-specific transcription factor OsSPL13 | Cell expansion | Si, et al. 2015 |
| *WG7* | a cysteine-tryptophan (CW) domain-containing transcriptional activator | Grain width | Huang, et al. 2020 |
| **Grain filling** |  |  |  |
| *GIF2* | An ADP‐glucose pyrophosphorylase (AGP) large subunit, AGPL2 | ADP-glucose synthesis | Wei, et al. 2017 |
| *GIF1* | Cell-wall invertase | Sucrose hydrolysis | Wang, et al. 2008 |

References:

Che, R., Tong, H., Shi, B., Liu, Y., Fang. S, [Liu](https://www.nature.com/articles/nplants2015195/#auth-6), D., [Xiao](https://www.nature.com/articles/nplants2015195/#auth-7), Y., [Hu](https://www.nature.com/articles/nplants2015195/#auth-8), B., [Liu](https://www.nature.com/articles/nplants2015195/#auth-9), L., [Wang](https://www.nature.com/articles/nplants2015195/#auth-10), H., [Zhao](https://www.nature.com/articles/nplants2015195/#auth-11), M. and [Chu](https://www.nature.com/articles/nplants2015195/#auth-12), C. (2015) Control of grain size and rice yield by GL2-mediated brassinosteroid responses. *Nat. Plants*, **2**, 15195.

[Chun, Y](https://www.ncbi.nlm.nih.gov/pubmed/?term=Chun%20Y%5bAuthor%5d&cauthor=true&cauthor_uid=32006119)., [Fangm J](https://www.ncbi.nlm.nih.gov/pubmed/?term=Fang%20J%5bAuthor%5d&cauthor=true&cauthor_uid=32006119), [Zafar, S.A](https://www.ncbi.nlm.nih.gov/pubmed/?term=Zafar%20SA%5bAuthor%5d&cauthor=true&cauthor_uid=32006119)., [Shang, J](https://www.ncbi.nlm.nih.gov/pubmed/?term=Shang%20J%5bAuthor%5d&cauthor=true&cauthor_uid=32006119)., [Zhao, J](https://www.ncbi.nlm.nih.gov/pubmed/?term=Zhao%20J%5bAuthor%5d&cauthor=true&cauthor_uid=32006119)., [Yuan, S](https://www.ncbi.nlm.nih.gov/pubmed/?term=Yuan%20S%5bAuthor%5d&cauthor=true&cauthor_uid=32006119). and [Li, X](https://www.ncbi.nlm.nih.gov/pubmed/?term=Li%20X%5bAuthor%5d&cauthor=true&cauthor_uid=32006119). (2020) *MINI SEED 2* (*MIS2*) Encodes a Receptor-like Kinase that Controls Grain Size and Shape in Rice. [*Rice*](https://www.ncbi.nlm.nih.gov/pubmed/32006119), **13**(1):7. doi: 10.1186/s12284-020-0368-9.

Fan, C., Xing, Y., Mao, H., Lu, T., Han, B., Xu, C., Li, X. and Zhang, Q. (2006) *GS3,* a major QTL for grain length and weight and minor QTL for grain width and thickness in rice, encodes a putative transmembrane protein. *Theor. Appl. Genet.* **112**, 1164–1171.

Guo, T., Chen, K., Dong, N.Q., Shi, C.L., Ye, W.W., Gao, J.P., Shan, J.X. and Lin, H.X. (2018) GRAIN SIZE AND NUMBER1 negatively regulates the OsMKKK10-OsMKK4-OsMPK6 cascade to coordinate the trade-off between grain NUMBER per panicle and grain size in rice. *Plant Cell*, **30**:871–888.

Heang, D. and Sassa, H. (2012) Antagonistic actions of HLH/bHLH proteins are involved in grain length and weight in rice. *PLoS ONE* **7**:e31325. https ://doi.org/10.1371/journ al.pone.00313 25.

Hu, Z., He, H., Zhang, S., Sun, F., Xin, X., Wang, W., Qian, X., Yang, J. and Luo X. (2012) A Kelch motif-containing serine/threonine protein phosphatase determines the large grain QTL trait in rice. *J. Integr. Plant Biol.* **54**:979–990.

Hu, J., Wang, Y., Fang, Y., Zeng, L., Xu, J.,  [Yu, H](https://www.ncbi.nlm.nih.gov/pubmed/?term=Yu%20H%5bAuthor%5d&cauthor=true&cauthor_uid=26187814)., [Shi, Z](https://www.ncbi.nlm.nih.gov/pubmed/?term=Shi%20Z%5bAuthor%5d&cauthor=true&cauthor_uid=26187814)., [Pan, J](https://www.ncbi.nlm.nih.gov/pubmed/?term=Pan%20J%5bAuthor%5d&cauthor=true&cauthor_uid=26187814)., [Zhang, D](https://www.ncbi.nlm.nih.gov/pubmed/?term=Zhang%20D%5bAuthor%5d&cauthor=true&cauthor_uid=26187814)., [Kang, S](https://www.ncbi.nlm.nih.gov/pubmed/?term=Kang%20S%5bAuthor%5d&cauthor=true&cauthor_uid=26187814)., [Zhu, L](https://www.ncbi.nlm.nih.gov/pubmed/?term=Zhu%20L%5bAuthor%5d&cauthor=true&cauthor_uid=26187814)., [Dong, G](https://www.ncbi.nlm.nih.gov/pubmed/?term=Dong%20G%5bAuthor%5d&cauthor=true&cauthor_uid=26187814)., [Guo, L](https://www.ncbi.nlm.nih.gov/pubmed/?term=Guo%20L%5bAuthor%5d&cauthor=true&cauthor_uid=26187814)., [Zeng, D](https://www.ncbi.nlm.nih.gov/pubmed/?term=Zeng%20D%5bAuthor%5d&cauthor=true&cauthor_uid=26187814)., [Zhang, G](https://www.ncbi.nlm.nih.gov/pubmed/?term=Zhang%20G%5bAuthor%5d&cauthor=true&cauthor_uid=26187814)., [Xie, L](https://www.ncbi.nlm.nih.gov/pubmed/?term=Xie%20L%5bAuthor%5d&cauthor=true&cauthor_uid=26187814)., [Xiong, G](https://www.ncbi.nlm.nih.gov/pubmed/?term=Xiong%20G%5bAuthor%5d&cauthor=true&cauthor_uid=26187814)., [Li, J](https://www.ncbi.nlm.nih.gov/pubmed/?term=Li%20J%5bAuthor%5d&cauthor=true&cauthor_uid=26187814). and [Qian, Q](https://www.ncbi.nlm.nih.gov/pubmed/?term=Qian%20Q%5bAuthor%5d&cauthor=true&cauthor_uid=26187814). (2015). A rare allele of *GS2* enhances grain size and grain yield in rice. *Mol. Plant*, **8**, 1455–65.

Huang, X., Qian, Q., Liu, Z., Sun, H., He, S., Luo, D., Xia, G., Chu, C., Li, J. and Fu, X. (2009) Natural variation at the *DEP1* locus enhances grain yield in rice. *Nat. Genet.* **41**:494–497.

Huang, Y., Bai, X., Cheng, N., Xiao, J., Li, X. and Xing, Y. (2020) *Wide Grain 7* increases grain width by enhancing H3K4me3 enrichment in the *OsMADS1* promoter in rice (*Oryza sativa* L.). *Plant J*. doi: 10.1111/tpj.14646.

Ishimaru, K., Hirotsu, N., Madoka, Y., Murakami, N., Hara, N., Onodera, H., Kashiwagi, T., Ujiie, K., Shimizu, B., Onishi, A., Miyagawa, H. and Katoh, E. (2013) Loss of function of the IAA-glucose hydrolase gene *TGW6* enhances rice grain weight and increases yield. *Nat. Genet*. **45**, 707–11.

Jiang, Y., Bao, L., Jeong, S.Y., Kim, S.K., Xu, C., Li, X. and Zhang, Q. (2012) XIAO is involved in the control of organ size by contributing to the regulation of signaling and homeostasis of brassinosteroids and cell cycling in rice. *Plant J*. **70**, 398–408.

Li, Y., Fan, C., Xing, Y., Jiang, Y., Luo, L., Sun, L., Shao, D., Xu, C., Li, X., Xiao, J., He, Y. and Zhang, Q. (2011) Natural variation in GS5 plays an important role in regulating grain size and yield in rice. *Nat. Genet.* **43**:1266–1269.

Liu, L., Tong, H., Xiao, Y., Che, R., Xu, F., Hu, B., Liang, C., Chu, J., Li, J. and Chu C.(2015) Activation of *Big Grain1* significantly improves grain size by regulating auxin transport in rice. *Proc. Natl. Acad. Sci. USA.* **112**:11102–11107.

Liu, J., Chen, J., Zheng, X., Wu, F., Lin, Q., Heng, Y., Tian, P., Cheng, Z., Yu, X., Zhou, K., Zhang, X., Guo, X., Wang, J., Wang, H. and Wan, J. (2017) *GW5* acts in the brassinosteroid signalling pathway to regulate grain width and weight in rice. *Nat. Plants,* **3**,17043.

Lo, S.-F., Cheng, M.-L., Hsing, Y.-I., Chen, Y.-S., Lee, K.-W., Hong, Y.-F., Hsiao, Y., Hsiao, A.-S., Chen, P.-J., Wong, L.-I., Chen, N.-C., Reuzeau, C., Ho, T.-H. D.and Yu, S.-M. (2020) Rice Big Grain 1 promotes cell division to enhance organ development, stress tolerance and grain yield. *Plant Biotechnol. J*. <https://doi.org/10.1111/pbi.13357>.

Luo, J., Liu, H., Zhou, T., Gu, B., Huang, X., Shangguan, Y., Zhu, J., Li, Y., Zhao, Y., Wang, Y., Zhao, Q., Wang, A., Wang, Z., Sang, T., Wang, Z. and Han, B. (2013) *An-1* encodes a basic helix-loop-helix protein that regulates awn development, grain size, and grain number in rice. *Plant Cell,* **25**, 3360–76.

Nakagawa, H., Tanaka, A., Tanabata, T., Ohtake, M., Fujioka, S., Nakamura, H., Ichikawa, H. et al. (2012) Short grain1 decreases organ elongation and brassinosteroid response in rice. *Plant Physiol*.**158**: 1208–1219.

Qi, P., Lin, Y., Song, X., Shen, J., Huang, W., Shan, J., Zhu, M., Jiang, L., Gao, J. and Lin, H. (2012) The novel quantitative trait locus *GL3.1* controls rice grain size and yield by regulating Cyclin-T1;3. *Cell Res*. **22**, 1666–1680.

Si L, Chen J, Huang X, Gong H, Luo J, Hou Q, Zhou T, Lu T, Zhu J, Shangguan Y, Chen E, Gong C, Zhao Q, Jing Y, Zhao Y, Li Y, Cui L, Fan D, Lu Y, Weng Q, Wang Y, Zhan Q, Liu K, Wei X, An K, An G, Han B (2015) OsSPL13 controls grain size in cultivated rice. *Nat. Genet.* **48**:447–456.

Song, X., Huang, W., Shi, M., Zhu, M. and Lin, H. (2007) A QTL for rice grain width and weight encodes a previously unknown RING-type E3 ubiquitin ligase. *Nat. Genet*. **39**, 623–30.

Utsunomiya, Y., Samejima, C., Takayanagi, Y., Izawa, Y., Yoshida, T., Sawada, Y., Fujisawa, Y., Kato, H. and Iwasaki, Y. (2011) Suppression of the rice heterotrimeric G protein beta-subunit gene, *RGB1*, causes dwarfism and browning of internodes and lamina joint regions. *Plant J*. **67**, 907–916.

Wang, E., Wang, J., Zhu, X., Hao, W., Wang, L., Li, Q., Zhang, L., He, W., Lu, B., Lin, H., Ma, H., Zhnag, G. and He, Z. (2008) Control of rice grain-filling and yield by a gene with a potential signature of domestication. *Nat. Genet*. **40**, 1370–1374.

Wang, S., Li, S., Liu, Q., Wu, K., Zhang, J., Wang, S., Wang, Y., Chen, X., Zhang, Y., Gao, C., Wang F., Huang, H. and Fu, X. (2015) The *OsSPL16-GW7* regulatory module determines grain shape and simultaneously improves rice yield and grain quality. *Nat. Genet*. **47**, 949–54.

Wei, X., Jiao, G., Lin, H., Sheng, Z., Shao, G., Xie, L., Tang, S., Xu, Q. and Hu, P. (2017) [*GRAIN INCOMPLETE FILLING* 2 regulates grain filling and starch synthesis during rice caryopsis development.](https://www.ncbi.nlm.nih.gov/pubmed/27957808) *J. Integr. Plant Biol*. **59**, 134-153.

Zhong, J., He, W., Peng, Z., Zhang, H., Li, F. and Yao, J. (2020) A putative AGO protein, OsAGO17, positively regulates grain size and grain weight through OsmiR397b in rice. *Plant Biotechnol. J*. **18**, 916–928.

Zhou, Y., Zhu, J., Li, Z., Yi, C., Liu, J., Zhang, H., Tang, S., Gu, M. and Liang, G. (2009) Deletion in a quantitative trait gene *qPE9-1* associated with panicle erectness improves plant architecture during rice domestication. *Genetics,* **183**, 315–324.
