# Supplementary figures and images for: RGB1 Regulates Grain Development and Starch Accumulation Through Its Effect on OsYUC11-Mediated Auxin Biosynthesis in Rice Endosperm Cells
Source: Front Plant Sci. 2021 Mar 31;12:585174. doi: 10.3389/fpls.2021.585174 (PMC8045708; doi:10.3389/fpls.2021.585174)

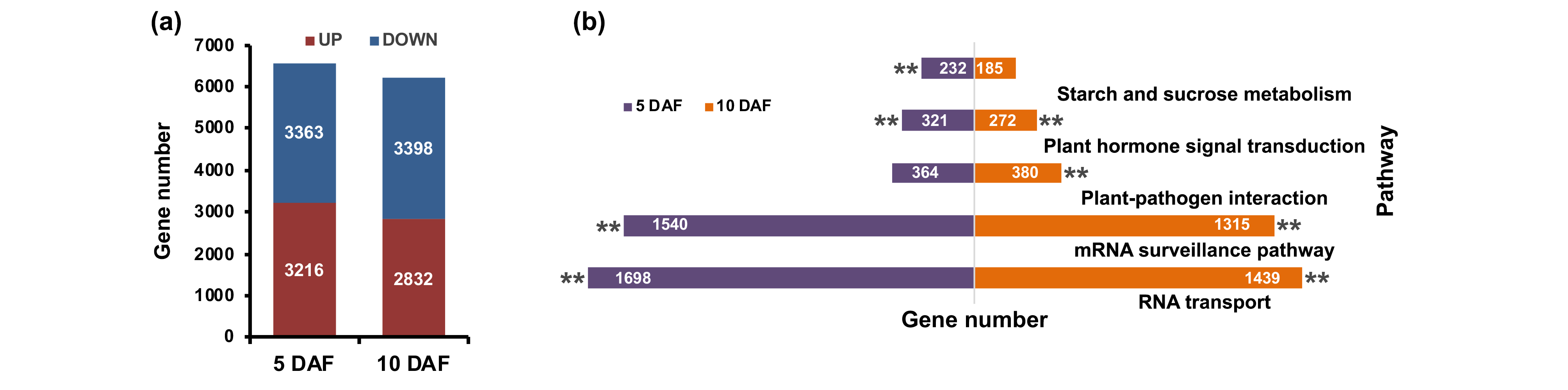

Supplement: Supplementary file 3 [file Image_1.JPEG]

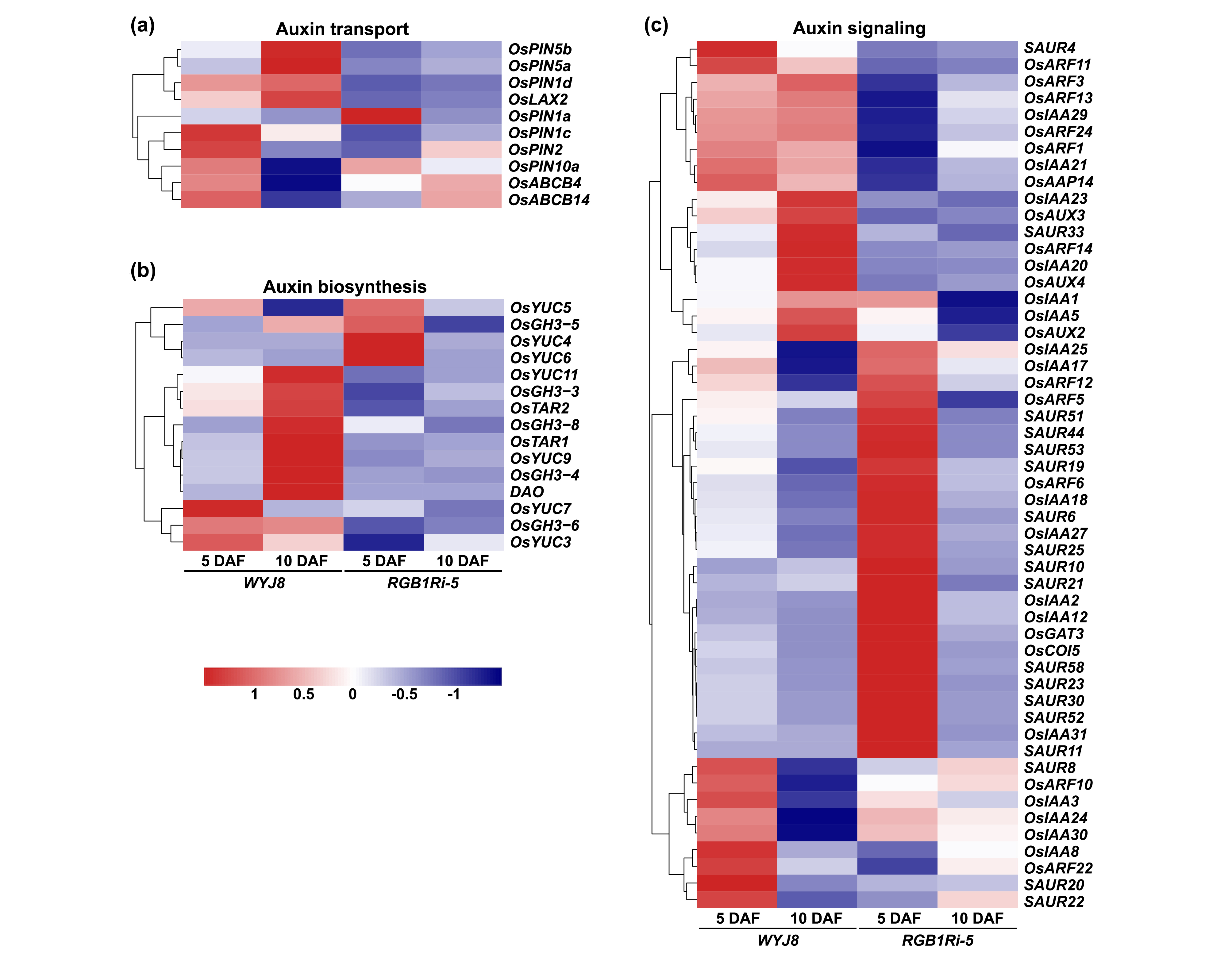

Supplement: Supplementary file 4 [file Image_2.JPEG]

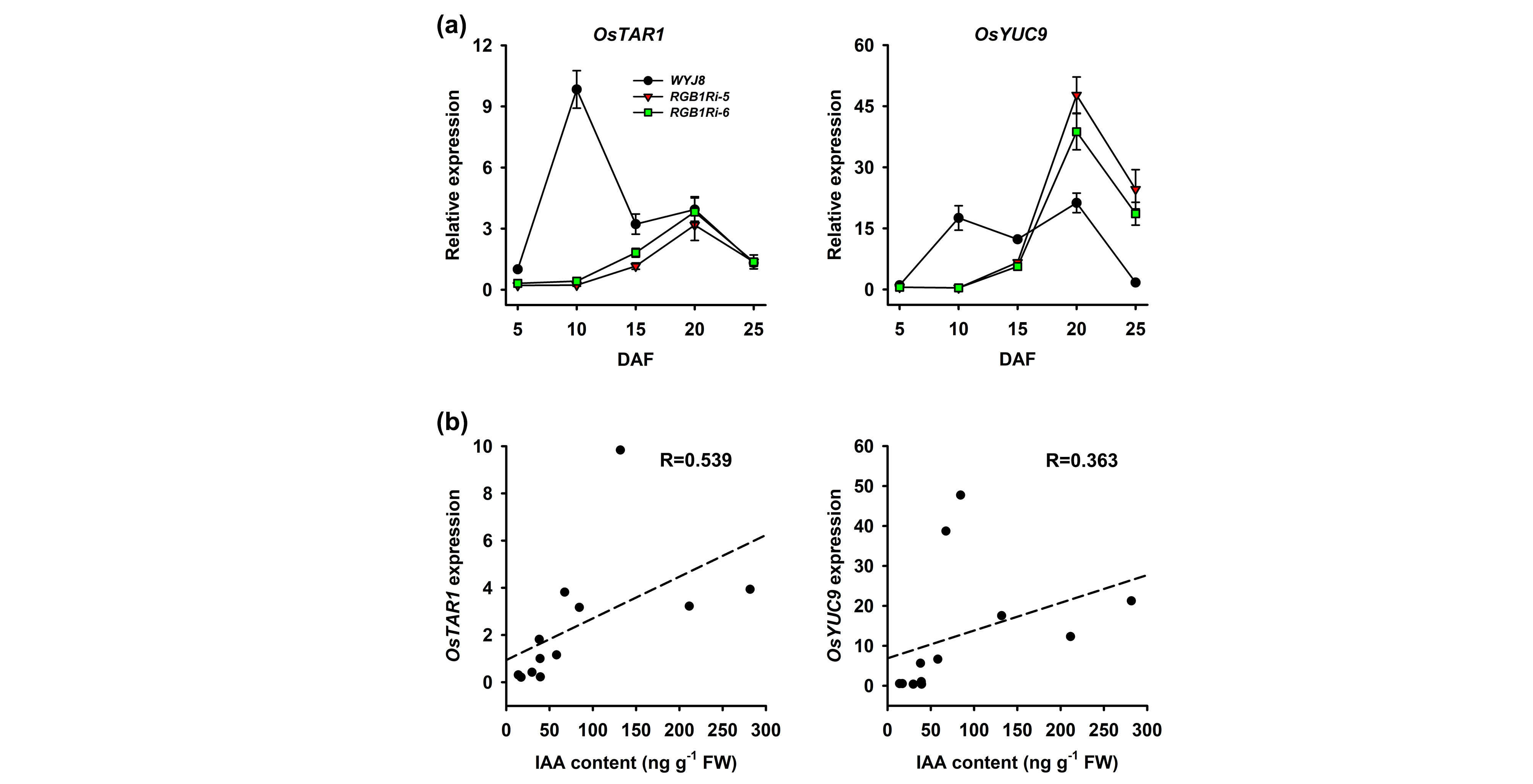

Supplement: Supplementary file 5 [file Image_3.JPEG]
